# ‘From Superman to Barely Can:’ The Experience of Ageing for Australians With Spinal Cord Injury Sustained Below the Age of 65—A Qualitative Study

**DOI:** 10.1111/hex.70787

**Published:** 2026-07-28

**Authors:** Emma Tan, Lynette Mackenzie, Jacqueline Wesson, Shane Clifton

**Affiliations:** ^1^ Discipline of Occupational Therapy, Sydney School of Health Sciences, Faculty of Medicine and Health The University of Sydney Sydney New South Wales Australia; ^2^ The Centre for Disability Research and Policy, Sydney School of Health Sciences, Faculty of Medicine and Health The University of Sydney Sydney New South Wales Australia

**Keywords:** advocacy, ageing, occupational therapy, participation, spinal cord injury (SCI), support services

## Abstract

**Background:**

Although ageing is universal, spinal cord injury (SCI) intensifies age‐related physical, psychosocial, cognitive, and functional challenges, with greater consequences for independence and quality of life. Improved life expectancy exposes limitations in prevailing ‘ageing well’ frameworks that exclude disability.

**Methods:**

This qualitative study used a phenomenological approach. Semi‐structured interviews were conducted online with community‐dwelling adults aged ≥ 50 years who sustained SCI before age 65 and at least 2 years previously, and carers of people who met these criteria. Interviews were recorded, transcribed verbatim, and analysed thematically.

**Results:**

Twenty‐five interviews were completed (22 people with SCI and 3 carers). Participants described ageing as inevitable but distinctly shaped by SCI. Three themes captured their experiences: (1) *Compounding vulnerabilities: the experience of accelerated ageing*, describing progressive bodily change, health instability, and adjustment to ageing; (2) *Converging impacts of biological change and policy gaps*, highlighting cumulative functional impacts alongside challenges navigating services and environmental and attitudinal barriers; and (3) *Ageing with meaning through participation*, emphasising agency, self‐advocacy, and maintaining engagement in work, leisure, and social roles. Adequate disability funding and health and system literacy supported participation, whereas limited SCI‐specific knowledge within health and care services and resource constraints undermined ageing well.

**Conclusions:**

Ageing well with SCI is shaped by individual adaptation and modifiable system‐level factors. Policies that ensure equitable resourcing, strengthen ageing (including SCI‐competent services), and support meaningful participation may improve quality of life as this population grows.

**Lived Experience or Public Contribution:**

People with lived experience of spinal cord injury were actively involved throughout the research process. A consumer advisory panel (CAP) was established following a national expression‐of‐interest process and comprised eight individuals with lived experience, one carer and a researcher with lived experience. The CAP contributed to study design, recruitment strategies, and interpretation and validation of findings, and supported aspects of data analysis. Panel members represented diverse injury characteristics, time since injury and environmental contexts, enabling identification and consideration of assumptions and biases. In addition, people with lived experience participated as interviewees in the study.

**Trial Registration:**

This study was not a clinical trial.

## 1 Introduction

‘From superman to barely can,’ is a song lyric describing deterioration with ageing. A study participant who sustained a spinal cord injury (SCI) earlier in life used this lyric to express the frustration of losing previously gained independence as he gets older.

Everyone ages, and there are commonalities in the ageing process for those with SCI and those without. However, the physical, functional, psychosocial, cognitive and emotional effects of SCI exacerbate general concerns, such as functional independence, and engagement in social, leisure, and work activities, with more significant consequences for quality of life and independence.

Historically, life expectancy for people with SCI was limited, leaving a significant gap in evidence about their experiences [1]. Although advances in medical treatment and rehabilitation mean they are increasingly living into older age [2, 3], prevailing definitions of ‘ageing well’ continue to specify ‘ageing without disability or disease’ [4], effectively excluding people with SCI [5]. Adopting a more inclusive approach prioritises quality of life for all people as they age, regardless of physical function [6]. Research into ageing with SCI is now imperative as it will help to identify the social and economic strategies required to support and optimise quality of life.

A previous scoping review indicated that the experience of ageing differs depending on whether the injury is sustained earlier or later in life [7]. When an SCI is sustained earlier in life, people have already adapted to functional changes before experiencing age‐related changes later in life: their experience of ageing with SCI is different. In addition, in Australia, the National Disability Insurance Scheme (NDIS) supports people injured below 65 years, while those who are older (> 65 years) are supported by the aged care system [8]. The financial support provided by these two schemes differs markedly, potentially affecting the experience of ageing, challenges encountered, and long‐term outcomes. This paper focuses on the experiences of people who sustained their injury before age 65 who are ageing *with SCI* (as part of a broader project on this topic). It aims to identify how ageing affects physical, cognitive and psychosocial perspectives, and how this impacts their ability to ‘age well’, including supports currently available and any gaps.

## 2 Methods

The study was conducted by a four‐member research team. Author 1 (female), an occupational therapist and PhD candidate, contributed clinical knowledge from occupational therapy practice in spinal cord injury and ageing. Authors 2 (female) and 3 (female) brought academic expertise in ageing‐focused occupational therapy research, with Author 3 also drawing on prior clinical work with older adults. Author 4 (male), an academic, added expertise in disability research and policy, alongside lived experience of spinal cord injury. Lived experience insights from Author 4 and the Consumer Advisory Panel (CAP) informed the study's development, interpretation, and reflexive analytical processes.

### 2.1 Consumer Advisory Panel

At the start of the broader project, a national expression‐of‐interest process identified volunteers with lived experience who were interested in the research focus. A consumer advisory panel (CAP) of eight volunteers with lived experience, one carer, and a research team member with lived experience was established to provide advice, ensure relevance and validate results. Panel participants had different SCI levels and severities, time since injury and environmental contexts (see Supporting Information S1: Appendix 1). The diversity of demographic backgrounds ensured that any assumptions and biases could be identified and considered.

### 2.2 Study Design

This qualitative study used online semi‐structured interviews with people with lived experience of ageing who acquired their SCI under the age of 65. The study explores knowledge gaps identified from a previous scoping review, including experiences of cognitive decline, residential aged care, and adjustment to ageing with SCI [7].

Ethics approval was provided by the University of Sydney Human Research Ethics Committee (2024/HE000461).

### 2.3 Aims

The study aims to identify (1) how people ageing with SCI sustained under the age of 65 and their carers perceive the concept of ‘ageing well’, including the supports currently available and any gaps, (2) how ageing affects this group from physical, cognitive and psychosocial perspectives, and (3) their challenges specifically related to ageing.

### 2.4 Recruitment

#### 2.4.1 Eligibility Criteria

The eligibility criteria to participate were as follows:

i. Participants with SCI: have sustained their SCI below the age of 65 and more than 2 years prior, and be above the age of 50 years old (to account for accelerated ageing in this cohort)
ii. Participants who are carers (any age) of a person who fulfils the above criteria.

All participants were living in the community (either in private accommodation, disability accommodation or residential aged care) and were fluent in written and verbal English.

#### 2.4.2 Recruitment of Participants

Participants were recruited through promotional materials distributed via consumer groups, primary health networks and professional organisations and through snowballing. Participants reviewed the study aims and purpose, including information about the research teams’ academic affiliations and qualifications. Participants then expressed interest and provided consent via an online Qualtrics form [9].

### 2.5 Data Collection

i. The interview questions used for online data collection were based on gaps in knowledge identified through the scoping review [7] and in collaboration with the CAP, discussed during a virtual group discussion (see Supporting Information S1: Appendix 2).
ii. Author 1 conducted semi‐structured interviews with people with lived experience and carers, with Author 3 also attending some. Interviews were of approximately 1 h duration. Interviews occurred via Zoom [10] to enable people from diverse backgrounds, locations and with mobility restrictions to participate. Participants were given the option of having a support person present, but none selected this option.
iii. Interviews were recorded (both audio and visual) and transcribed with field notes made during and post‐interview. No repeat interviews were conducted.
iv. Transcriptions were coded using NVivo [11] and analysed using thematic analysis underpinned by a phenomenological approach, with themes derived from these codes. Author 1 coded all the interviews with Authors 2, 3 and 4 coding a subset each, which were then compared to ensure consistency and consensus. Interviews were conducted iteratively until data saturation was reached. Interview transcripts and results were discussed with the CAP, who provided feedback and recommendations in a further two meetings.

The COREQ reporting guideline and checklist [12] were used to guide this manuscript (see Supporting Information S1: Appendix 3).

## 3 Results

Twenty‐two people with SCI sustained below the age of 65 and three carers participated. People with SCI were predominantly male (72.7%), with average age of 63.1 (±7.7, 53–81) and average time since injury of 29.5 years (±15.9, 4.5–61.0) and all carers were female. Eleven participants had tetraplegic SCI, with 10 participants with paraplegia. The majority of participants sustained their SCI through traumatic mechanisms, with only one acquiring a non‐traumatic injury (due to a spinal abscess). Most participants lived with a partner or family member, seven lived alone but were supported by formal care. All participants described strong social, and community support networks. Most were from metropolitan areas (59.1%), with representation from regional (13.6%), rural (22.7%) and remote contexts (4.5%), as classified by the Modified Monash Model [13]. The participants’ geographical diversity highlights the inclusive study methodology, which supported participation of those with mobility impairments or in remote locations by removing accessibility barriers.

While the perspectives of people with lived experience and their carers were largely consistent regarding the factors shaping the experience of ageing, some differences were also identified. People with lived experience focused on independence, participation, adjustment, and identity, whereas carers placed greater emphasis on recognising age‐related changes and required adaptations, planning for future needs, the sustainability of support, and how ageing affected their relationship with the person they were caring for.

Three key themes were identified through the interviews: ‘compounding vulnerabilities: the experience of accelerated ageing’, ‘converging impacts of biological change and policy gaps’, and ‘ageing with meaning through participation’. Quotes have been used to illustrate these themes, with the code ‘LE’ indicating a ‘lived experience’ participant, and ‘CAR’ indicating a carer.

### 3.1 Theme 1: Compounding Vulnerabilities: The Experience of Accelerated Ageing

Participants described their experiences of physical and functional decline as they aged, how this was ‘accelerated’ for those with SCI and their adjustment to these changes.

#### 3.1.1 What Does Ageing Look Like for People With SCI?

Participants recognised functional decline (fatigue, osteoporosis, falls) as typical of ageing, but more severe with SCI. They emphasised that although ‘wear and tear’ occurs in all ageing bodies, SCI necessitates sustained upper‐limb use for tasks they are not designed for. Long‐term upper‐limb loading for wheelchair propulsion and transfers leads to overuse injuries (e.g., rotator cuff tears, upper limb/neck arthritis, carpal tunnel syndrome), often requiring surgery and lengthy rehabilitation, reducing independence, increasing care needs and impacting broader life roles. As one participant observed:

Your body seems to age prematurely, and that's because of all the overuse … I had to retire from work early.

(LE16)

Participants described the interaction between ageing and SCI‐related secondary conditions as ‘accelerated ageing’, with earlier and faster decline than able‐bodied peers, limiting meaningful activity and challenging adjustment to reduced independence. One participant noted:

It [functional decline]…was gradual. But as I aged it went into zoom mode… Yeah, in the last 6 years. Oh, unbelievable!‥

(LE12)

Pain was common and could be compounded due to the cumulative effects of SCI‐ and age‐related pain, but impaired sensation altered how pain was detected, sometimes presenting via autonomic symptoms (e.g., increased blood pressure, sweating, headaches), consistent with autonomic dysreflexia, complicating assessment and diagnosis. Participants discussed a range of strategies to manage pain, including medication, changing position, reducing time sitting up in their wheelchairs and distractions through other activities. One participant described their level of pain as, ‘fairly high…I guess I've learned and am learning how to deal with chronic pain’ (LE8).

Weight gain can be a product of SCI, particularly for people with power wheelchairs, who cannot get the exercise equivalent of nondisabled people, with amplified functional consequences, such as increasing difficulty with transfers and mobility and prompting equipment changes or additional carers. It also has secondary consequences and other aspects of health, including heart disease and impacts on other organs, and fatigue from being unfit. Age‐related skin fragility, reduced sensation, and mobility impairment increased pressure injury risk, with prolonged wound healing, sometimes necessitating extended bed rest.

Participants described challenges with bladder and bowel management as they aged, ‘I self‐catheterised, for I think it was 28 years. It just got too hard to get on and off the toilet all the time, so I got an SPC (suprapubic catheter)’ (LE9). Bowel and bladder care became more time‐consuming and more difficult due to functional and physiological changes, and complications such as increased urinary tract infections. As a result, strategies such as colostomy and suprapubic or indwelling catheters were implemented, with positive outcomes.

Participants reported heightened vulnerability and potential rapid deterioration due to the combination of SCI, age‐related factors, and comorbidities, with one participant describing:

You are aware of your vulnerability and that things can go pear shaped fairly quickly with a high‐level SCI….through a chest infection‥ skin breakdown… catheter care and autonomic dysreflexia.

(LE3)

These factors may contribute to an earlier death, an existential reality that those with SCI must face. Many participants anticipated self‐identified needs and implemented strategies through experience, research, and trial and error, often without clinician input; over time, they reported greater confidence, including reducing medication, using alternative therapies, yoga, and marijuana.

#### 3.1.2 How Do People Respond to These Changes?

Participants reported that they initially did not anticipate ‘that it [ageing] would make a difference’ (LE9), largely because many had not expected to live long enough to experience age‐related change.

One participant reflected on sub‐optimal medical decisions made at the time of injury, based on assumptions that they would not survive beyond adolescence. As a result, many participants were unaware of, and unprepared for, age‐related changes, making later adjustment particularly confronting.

Ageing with SCI was associated with grief, loss, and identity disruption as participants adapted to changes in functional capacity and life roles. Retirement, while challenging for many older adults, was described as more complex when driven by functional deterioration rather than choice, particularly when physical limitations restricted participation in activities commonly associated with retirement. While adjusting to the initial SCI was described as difficult, it often involved a more discrete and clearly defined functional change that participants eventually learned to accommodate. In contrast, ageing was characterised by gradual, cumulative decline and ongoing uncertainty, which participants found more challenging to adapt to emotionally and practically. As one participant noted, ‘I struggle with this more than I struggled with the onset of the injury’ (LE12).

Adjustment to ageing varied, with three broad approaches: (i) denial, in which participants avoided information about ageing because it threatened hard‐won independence; some ignored changes until a crisis (e.g., hospitalisation) necessitated acceptance; (ii) ‘gradual surrender’, characterised by ongoing adaptation to emerging challenges cumulatively; and (iii) moving towards acceptance, often involving recognition that quality of life could improve with supports and environmental modifications that enabled function and participation, and taking agency to make change. However, accepting increased care remained confronting for some, depending on the level of support previously required, leading one participant to reflect:

It's like a gradual surrendering to reality. You know… I can fight this as much as I want, but it doesn't help…I've always been pretty fiercely independent. So that … surrendering independence at whatever level, doesn't come easy to me.

(LE8)

Over time, many participants described a shift in how they conceptualised independence. Whereas independence was initially defined as performing tasks with little or no assistance, it was later reframed as maintaining autonomy in decision‐making and sustaining participation in meaningful roles and activities. In this context, participants recognised that accepting practical support was described not as a loss of independence but as a means of conserving energy, reducing risk, and enabling engagement in valued activities:

I'm so much more dependent now which I don't like, but I've accepted because I feel very independent in my life…I have to accept the help to enable me to continue to do all the things that I really like to do.

(LE9)

This was also demonstrated by the evolving ways participants used assistive equipment, such as wheelchairs. Many participants described having lived most of their lives in manual wheelchairs; however, as they aged, the physical demands of propelling and frequent transfers became more challenging. As a result, many now use a combination of manual and power wheelchairs depending on the situation. Others were using power assist to facilitate outdoor mobility. One participant reflected on this adjustment,

I had a power wheelchair years ago… and I did feel more self‐conscious in it… But that was when I was younger and feeling like I needed to prove that I was independent…Whereas that's less of an issue these days. You sort of grow more comfortable in your own skin.

(LE3)

Despite ongoing challenges, participants also reported positive psychological changes associated with ageing with SCI. Increased self‐acceptance, confidence, and resilience were commonly attributed to accumulated experience and perspective. Many described enhanced advocacy skills, including greater confidence in articulating needs and negotiating systems and environments, with one participant expressing:

I love being 53. I just feel much more my own person. Much clearer with my boundaries…… I wish it hadn't taken so long to get to this place of not feeling so vulnerable and so guilty about my needs.

(LE21)

Some participants characterised ‘age as a leveller’, noting that age‐related decline among able‐bodied peers reduced feelings of difference and, for some, fostered a sense of preparedness for ageing‐related change:

I look at old age…we're all gonna go through it, whether in a chair or not. Maybe mine's going to be a bit harder. But on the other hand, a lot of the adjustments people are going to have to make, I made at 35.

(LE19)

Maintaining a positive future orientation through goal‐setting and anticipating meaningful experiences was identified as a protective strategy that supported motivation and buffered against pessimism about future decline. One participant concluded:

I've got myself worked out…I can see what I'd like to do in the future. I've set myself goals. You gotta have something to look forward to. (LE1)

### 3.2 Theme 2: Converging Impacts of Biological Change and Policy Gaps

Participants described multiple factors that negatively shape ageing experiences, while noting gradual improvement as disability awareness and inclusion increase. They identified two key challenges: non‐inclusive government funding and services, and disempowering individual‐level barriers.

#### 3.2.1 Government Funding and Services Are Not Inclusive

Participants emphasised that adequate funding is essential for ageing well. Most received support through the NDIS or similar schemes such as iCare, which were generally perceived as enabling autonomy, choice, and control. Funding was allocated based on assessed need and directed towards priorities, including personal care, community access, employment, therapy to maintain function, assistive equipment, and home or vehicle modifications. Although overall funding levels were often viewed as sufficient, participants reported systemic inefficiencies, including lengthy approval processes, high documentation burden, insufficient allocations, inconsistent decision‐making, and difficulties navigating scheme complexity. Flexibility in plan management (NDIS‐managed, plan‐managed, or self‐managed) was highly valued, and participants generally felt they had meaningful control over how goals were resourced and funds spent, with one articulating:

It's individualised funding. You control the funding yourself. And you have much more freedom about how you use that funding in terms of the services that you seek.

(LE3)

While not directly affecting this cohort, participants raised concerns about systemic exclusion associated with the NDIS age cut‐off for individuals who sustain injury after age 65, which was perceived as discriminatory and misaligned with the needs of people ageing with lifelong disability.

NDIS funding supported ageing in place, a priority for most participants. As functional capacity declined, participants increasingly required assistance with personal care, domestic tasks, and community access. However, they stressed that support must be reliable, high‐quality, and delivered by workers with SCI‐specific expertise. Poor‐quality care, including worker negligence, was associated with serious adverse outcomes such as pressure injury, as described by one participant:

My wife did buddy shifts to show them how they should assist me to transfer. They ignored everything. They just rammed the slide board in.

(LE17)

Hospital services were also identified as critical to ageing well, yet participants described substantial barriers to appropriate care. With age, participants anticipated more frequent hospitalisation due to secondary conditions and comorbidities; however, hospitals were often perceived as poorly equipped to meet the needs of people with SCI. Barriers included environments that limited independence, inaccessible equipment, staff who did not listen, and limited SCI‐specific knowledge. These gaps were experienced as disempowering and, in severe cases, resulted in hospital‐acquired pressure injuries and iatrogenic disability. Participants also described dismissive clinical interactions in which professional judgement was prioritised over lived expertise, sometimes leading to harmful outcomes, for example:

They didn't know what autonomic dysreflexia is. I knew I had autonomic dysreflexia… And he [doctor] just gave me a blood pressure pill…one of my biggest fears is being able to trust the medical people because I've just heard so many horror stories.

(LE16)

Some perceived an age‐related shift in clinical focus towards maintenance rather than improvement of functional capacity, reinforcing feelings of devaluation with ageing. One participant described his observations:

So when you're a young person with disability…these are all your options ahead of you. And then, when you reach a certain age…they tend to treat you as… keeping your existence the status quo.

(LE17)

Participants accessed a range of community‐based medical and allied health services but reported persistent access barriers, including limited availability of skilled clinicians, physical inaccessibility of clinics, long waitlists, inadequate transport support, funding ineligibility for some therapies, and loss of continuity when trusted providers retired or relocated. These barriers reduced access to trusted care and increased disengagement risk, reinforcing perceptions that health systems were not designed to support ageing with disability. Participants valued collaborative relationships with general practitioners (GPs) who listened to and learned from lived experience despite limited SCI knowledge, with one explaining:

So I go to a GP. They generally don't know a lot about SCI. So there's a bit of an education process that happens within that they go off and do their research. And so if they're a good GP, they will listen…to what that person's got to say.

(LE8)

Multidisciplinary SCI clinics were also viewed positively, although access was restricted due to metropolitan concentration and limited outreach services.

Access to skilled services was further constrained for participants living in rural or regional areas. Clinicians were more often generalists with limited SCI expertise, hospitals lacked adequate resources to support people with SCI, and recruiting support workers was difficult. In these contexts, funding was irrelevant if services or workers were unavailable. Limited access to specialist services and inaccessible transport and infrastructure contributed to increased isolation. As one rural participant noted:

out here… it might be one paediatric OT….For gardening or home services, you're only looking at about 4 (providers). No social workers…no service providers for assistive technology…

(LE16)

Housing accessibility and security also shaped ageing experiences. While accessible housing supported independence, it was difficult to obtain, with one participant reflecting, ‘I didn't realise how hard people are finding it to find suitable accommodation as they age…or whether they can stay in their own home, especially people that are injured later in life’ (LE16). Some participants relocated to retirement villages, which were viewed positively for balancing independence with access to care and social support, although high entry costs were a barrier. Relocation decisions were often driven by safety, proximity to family, access to assistance, and climate stability related to thermoregulation.

Across social, health, and disability systems, participants reported experiencing discrimination, most commonly disability‐based rather than age‐based, although both were evident. Ignorance‐based discrimination included assumptions that physical disability implied cognitive impairment, while passive discrimination reflected limited SCI knowledge, poor accessibility, and misuse of accessible facilities such as toilets and parking spaces, restricting participation. Examples included:

You're in a wheelchair. So some people…they bend down and speak to you like you've got no brain. And it's so misunderstood…. Everyone thinks that if you're paraplegic, you're a cripple in the corner, with dribble running from your mouth.

(LE18)

Participants also described internalised ableism, which influenced their adjustment to disability and acceptance of age‐related functional changes. Many spoke of having to advocate and fight for equality throughout their lifetimes and reaching a stage of fatigue and resignation as they age, leading one participant to express, *‘*I'm just tired. I would love to just be able to get out the front door and do what every other Joe does without having to fight for it, you know’ (LE19).

#### 3.2.2 Individual Challenges Can Be Disempowering

Participants described fear of the future, ‘the kind of great unknown’ (LE7), driven by uncertainty about the trajectory and consequences of functional decline and loss of independence. Ongoing change led to frustration and emotional fatigue. As function declined, care needs often increased, and partners or family assumed greater support roles. Others reported role changes as they became caregivers for ageing relatives. Participants worried about the sustainability of informal care; some older participants required formal care following carer illness or death. One participant described their experience:

My mother got cancer. So superb, [mum] being my main carer here, living here with me. So that's the only reason we had to go on 24 hour care…‥ It's been a big change for me, though.

(LE1)

Although most had not experienced cognitive decline, participants feared age‐related cognitive impairment and its implications for autonomy. They emphasised that managing SCI requires continuous planning, problem‐solving, and self‐advocacy, and anticipated that cognitive impairment would have a disproportionate impact compared with able‐bodied ageing. As a participant explained:

Oh, and getting dementia… Because then you don't even have the capacity… and then you actually can't advocate for yourself as well… plus, I would forget to check my skin…

(LE16)

Many had pursued cognitively‐oriented roles (through work, leisure and advocacy) due to physical limitations and feared significant further restriction if cognition declined:

We all know somebody that's had dementia or Parkinson's… I've spent a lot of my life restricted through the body I've got. So I don't want that. I would hate to go through those sorts of things.

(LE14)

Participants reported worsening anxiety and depression with age, commonly linked to functional decline and reduced independence, and described ‘anticipatory anxiety’ about future loss of control. Coping strategies included staying active, seeking social support, and maintaining future‐oriented goals. Earlier adjustment to SCI shaped later ageing: acceptance and adaptive mindsets supported more positive ageing, whereas poor adjustment was linked to disengagement from communities, services, and participation.

Social isolation was viewed as an increasing risk due to reduced mobility and community access, relocation, fatigue, and the ageing or loss of support networks. As one person described:

Not one friend, not one relative, including my own children, made their place wheelchair accessible. So if they don't come to me, I don't see them.

(LE17)

Participants emphasised strong networks (partners, family, friends, employers, and peers with SCI) for connection and emotional support, with a lack of social networks significantly impacting emotional well‐being. Participants emphasised the importance of keeping spousal or family relationships distinct from care, ‘…don't ever let your partner or family do your personal care because it changes the relationship’ (LE9).

#### 3.2.3 Knowledge Is Empowering

Access to information about ageing with SCI is crucial to managing these challenges. Participants viewed ageing‐related information as empowering and essential for self‐advocacy. Self‐taught health and system literacy increased individuals with SCI and their primary carers’ confidence to ask questions, raise concerns, and make informed decisions by clarifying relevant options, resources, and supports. Participants argued that decisions should be respected when individuals understood the risks and consequences, even when clinicians disagreed:

…people are told that this is what aged care people do, and I don't want to do that. I want to come up with solutions that are more suitable for myself.

(LE10)

Peer support (informal and formal) was valued for emotional support, validation, and information sharing. Advice from peers with lived experience was often perceived as more credible and pragmatic than information from health professionals, as ‘…people who have the lived experience are far more helpful and knowledgeable…they have that lived experience’ (LE16).

However, participants reported limited ageing‐focused peer programs and a scarcity of mentors with lived experience of ageing, reflecting historically lower survival to older age. Only a minority received ageing‐related information from clinicians (ad‐hoc or targeted); most learned through observing others, social media, or online searches. Seeing others manage similar changes helped participants anticipate scenarios and consider options, ‘…it's a lot easier to visualise yourself doing something if you've seen someone with similar circumstances already doing it’ (LE2). Many wished they had been better prepared, yet noted initial rehabilitation typically prioritises short‐term goals. Suggested information provision methods are summarised in Supporting Information S1: Appendix 4.

Participants emphasised proactive health management to maintain quality of life, including acceptance, support, awareness of ageing impacts, and access to skilled services. Preventative strategies included diet, exercise, regular health checks, pacing, prioritising activities, and assistive equipment. Frequent health checks facilitate the identification of symptoms that may affect function and participation, enabling early intervention strategies.

### 3.3 Theme 3: Ageing With Meaning Through Participation

Despite these multiple challenges, participation was identified as central to ageing well. Participants described diverse activities that provided meaning and purpose, such as employment, advocacy, sport and creative pursuits, described in Supporting Information S1: Appendix 5.

#### 3.3.1 Participation Gives Life Meaning

Participation was prioritised by people ageing with SCI, particularly through contributing to their communities and ‘making a positive contribution to life’ (LE6).Many wished to share their life experiences and act as role models for family and the wider public, to ‘show people with a disability what they can do’ (LE6), demonstrating that disability is compatible with a meaningful life. Mentoring others was valued as ‘paying it forward’, reflecting gratitude for support previously received and a commitment to assisting others with SCI, ‘…if I can pass some of my knowledge on to a new paraplegic, my job is done’ (LE4). Participants emphasised that goals and purpose motivated active, productive living and enhanced fulfilment, ‘…you gotta have reasons why you get up every morning…Then you'll go and do it’ (LE1).

#### 3.3.2 What Occupations Bring Meaning for People With SCI as They Age?

Participants described finding meaning in a variety of occupations; however, they needed to develop adaptive strategies to maintain participation. Productive roles (employment, volunteering, study, advocacy) were highly valued for their purpose and social connection. As one participant expressed:

Spinal cord injury is my passion, because I live it and I can make changes that are going to improve somebody else's life or help educate.

(LE16)

Continuing to work often required adjustments (e.g., flexible hours, workplace adaptations), ‘I gradually went to part‐time and then home‐based’ (LE16); with limited employer flexibility and increasing difficulty contributing to early retirement for some. Participants also highlighted participation in self‐care and self‐management, ranging from independent task completion to directing carers. Age‐related functional decline affected mobility, transfers, self‐care, sleep, domestic activities and driving, with flow‐on effects on work and leisure. Adaptation strategies included pacing, planning and prioritisation, assistive technologies (e.g., hoists, power wheelchairs), and increased support. Leisure became increasingly salient approaching retirement but was constrained by declining function, reduced spontaneity due to scheduled care, and social exclusion linked to perceived planning burden. Travel aspirations were frequently limited by aviation and airport inaccessibility, sometimes leading to cessation of travel:

So there's all these kind of barriers, as I'm getting older, I'm…couldn't really be bothered. I did a lot of travelling by myself, and I never used to think twice about it, but I've done a couple of trips over the last couple of years by myself, and it's …. man, this is really hard work sorting out my luggage and getting in and out of different cars…and different bathrooms.

(LE8)

Participants were highly proactive in identifying assistive technology to sustain participation in meaningful roles. They described conducting extensive independent research into equipment that would facilitate participation, identifying innovative technologies (e.g., the ‘Genny’ gyroscopic mobility device), using assistive equipment in novel ways (e.g., using a bath lift to get back into their wheelchair after a fall at home), and making creative adaptations (such as a hand crank mechanism to propel a kayak).

Participants also described how transport was highly important to maintain participation. All participants utilised transport; some were driving, some could transfer into a vehicle to be driven, some used wheelchair‐accessible vehicles, and others used public transport. Many described how driving and transferring were becoming more difficult due to fatigue, pain, and reduced strength, utilising strategies such as pacing, having a carer drive, and using a slideboard to assist with transfers.

#### 3.3.3 The Nature of Participation Changes as People With SCI Age

Participation shifted with ageing, particularly due to fatigue, prompting more sedentary pursuits and greater planning to incorporate rest periods, ‘my A‐G‐E disease is gradually slowing me down’ (LE6). This created the risk of reduced participation in meaningful activities. Participants responded by prioritising social engagement and replacing physical activities with cognitively engaging alternatives, ‘…you do things with the brain’ (LE17). Others transitioned into advocacy or volunteering, drawing on lived experience to benefit others with SCI. Changing social roles also reshaped priorities, with greater emphasis on personally enjoyable and fulfilling roles after a lifetime of work:

I've felt that in my life I've always had a purpose, you know, a passion that I needed to pursue and now those sort of passions are not as important as my family…‥They are the things that are most important to me.

(LE10)

## 4 Discussion

**Figure 1:**
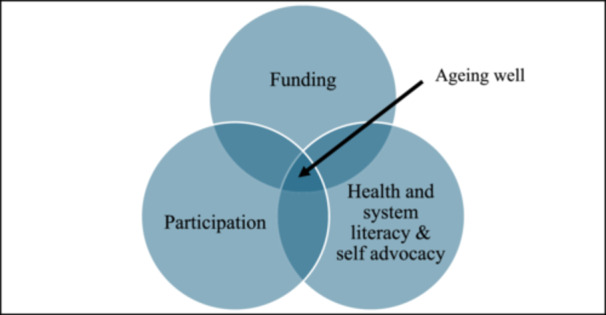
Key factors influencing ageing well for people who sustain their SCI below 65.

This study explores the experience of ‘ageing well’ for people who sustained their SCI below the age of 65, investigating what ageing looks like from physical, cognitive, and psychosocial perspectives and identifying current challenges, available supports, and gaps. Three crucial factors influencing the ability of people who sustained their SCI before age 65 to ‘age well’ were identified (Figure 1).

Funding was identified as the strongest determinant of ageing well with SCI in Australia. Where adequate funding was available, it enabled autonomy and access to the supports required to cover basic needs, live safely and remain engaged in the community [5]. In this context, funding functioned as an upstream enabler of other outcomes, including participation and quality of life.

These findings are consistent with Maslow's hierarchy of needs [14], which illustrates how people whose basic needs are met can then focus on self‐actualisation. Accordingly, participants demonstrated self‐actualisation through meaningful participation, which they viewed as central to quality of life as they age. Participation was associated with social connection, physical and cognitive challenge, and a sense of value [5, 15]. Many prioritised quality of life and meaning over longevity, describing life as of limited value without purpose. This is aligned with inclusive definitions of ageing well, demonstrating that people with SCI are actively ‘developing and maintaining the functional ability that enables well‐being in older age’ (World Health Organisation, 2020) [16]. In contrast, systems too often focus on the bottom of Maslow's hierarchy with comparatively less attention to higher‐order goals that sustain wellbeing. There was limited public, health professional, and service provider support for engaging in such activities [17], with self‐care and domestic tasks often prioritised by systems, overlooking meaningful leisure and participation goals [18].

Given the compounding challenges of ageing with SCI, these findings also emphasise self‐advocacy as a crucial protective capability. Health literacy is required to understand the health needs that must be negotiated to age well, whilst system literacy enables people to identify and navigate systems and processes that support ageing. Self‐advocacy then allows people to articulate and meet these needs [7]. Participants navigated multiple high‐level contexts: funding bodies, health and disability services, providers, and the public, and many undertook policy‐level advocacy through disability organisations and government advisory roles. People must also be able to advocate for themselves in their everyday lives, including communicating their needs to both formal and informal carers, providing training and guidance, and being assertive when they feel their needs are not being met [18]. It is imperative that people are empowered to advocate for themselves, especially since systems and poor understanding of SCI may contribute to negative health or safety outcomes [18].

Having information on ageing with SCI, including an understanding of policy and procedures, awareness of support structures, and knowledge of how others have approached ageing underpins health and system literacy. Provision of information facilitates self‐advocacy by enabling individuals to ask relevant questions, locate resources and allies, and challenge decisions when necessary. Information on ageing with SCI enhances people's sense of agency, empowering them to anticipate, adjust to, respond to, and accept change [7]. Having knowledge of ageing empowered participants to approach assistive technology as a proactive and positive strategy for sustaining participation in meaningful roles.

Peer support is a valuable way for consumers to access information [19]. Seeing someone with similar challenges leading a fulfilling life makes it easier for others to accept and adjust [7]. Peer support is also well established in SCI [20], however as people with lived experience were not previously reaching older age, there is a dearth of formal peer support mentors and programs to support those who are ageing [7]. Informal peer networks fill this gap.

Current community healthcare models for SCI are often reactive and not designed around ageing trajectories [7, 18]. These findings support the need for more proactive, ageing‐specific approaches, including regular multidisciplinary follow‐up to monitor functional change and secondary conditions associated with accelerated ageing [17, 21, 22, 23]. As more people with SCI live longer, an ageing‐specific model of care is increasingly necessary, consistent with approaches used in other diagnostic groups [24]. Without such models, people may disengage from services until a crisis or significant deterioration, limiting opportunities for prevention and early intervention [25]. This disengagement reflects both practical barriers, such as limited local services, travel constraints, and physical inaccessibility of clinics and hospitals [25], and limited SCI‐specific expertise among health professionals, which can lead to substandard care, impractical recommendations, and insufficient recognition of lived‐experience expertise [3, 23, 26, 27]. Hospital care warrants particular attention: admissions to settings without SCI capability may increase the risk of preventable harm, including loss of function and iatrogenic disability, with people with disability disproportionately represented in preventable hospital harm [28, 29]. By contrast, collaborative care, where clinicians acknowledge limits in their knowledge and actively incorporate lived expertise, appears to support stronger therapeutic relationships and safer, more effective care.

Geographical inequity further moderates access to ageing‐relevant healthcare and supports. Even when funding theoretically enables choice and control, this is constrained when skilled services are unavailable locally or require substantial travel [18]. This finding strengthens the rationale for more geographically equitable models of care (e.g., outreach, shared‐care arrangements, and service models that build rural capability), particularly as rural clinicians are less likely to have frequent exposure to SCI and related specialist resources [8].

These findings extend existing work on adjustment by highlighting ageing as a ‘second injury’, with the extent of decline being undefined and highly individual and requiring ongoing adaptation over time [30]. This aligns with the concept of ‘double jeopardy’ [31, 32], describing the compounding effects of disability and ageing. For those injured decades ago, adjustment to ageing also appears shaped by the historical context at their time of injury, where they needed to be self‐sufficient, pragmatic, and resourceful, yet had limited awareness of what to expect, including the possibility of age‐related functional decline. Having worked hard to achieve independence, confronting unexpected functional decline can be challenging, thereby complicating adjustment to ageing [7]. However, with adequate funding, health and system literacy and meaningful participation, people with SCI actively demonstrated the capacity to age well, consistent with contemporary inclusive definitions that recognise participation and quality of life rather than the absence of disability [6]. These findings underscore the importance of anticipatory guidance and ageing‐focused preparation earlier in the life course, rather than only once decline becomes unavoidable.

### 4.1 Strengths and Limitations

Participants in this study were generally well‐informed, proactive, and connected to advocacy networks. This was demonstrated by their preventative and positive approach to assistive technology to maintain participation. However, people who were more disengaged or socially isolated may have been underrepresented. Including these perspectives in future research could provide a more nuanced understanding of the drivers of disengagement and the types of supports that may facilitate re‐engagement. In addition, the sample did not include participants from culturally and linguistically diverse (CALD) backgrounds or Aboriginal and Torres Strait Islander peoples, which limits the cultural transferability of the findings to these populations. Only one participant acquired their SCI through a non‐traumatic mechanism, and this SCI was sustained later in life, reflecting evidence describing earlier life SCI as more likely to be through traumatic means, with non‐traumatic SCI more common among older adults [33]. As a result, perspectives of people with non‐traumatic SCI may also be under‐represented.

Strengths included the inclusive study method, with interviews conducted online. This allowed for participants with varying levels of mobility and from diverse geographical locations to participate, as demonstrated by the number of non‐metropolitan participants recruited. In addition, collaboration with the Consumer Advisory Panel ensured the relevance of the study objectives and design, and accuracy of interpretation of the results.

### 4.2 Study Implications

With many challenges affecting the experience of ageing, social policy that empowers people with SCI to engage in meaningful occupations through supports such as appropriate funding, information on ageing to enable informed decision‐making and self‐advocacy, and accessible and skilled support services is imperative. As the population of people ageing with SCI increases and they live longer, designing and implementing policies that facilitate productive participation and ageing well has significant individual, social, and economic benefits.

This study also identified implications for current theory on ageing with SCI. The findings extend the current understanding of adjustment, conceptualising it as a dynamic, ongoing process spanning the lifespan, in which people adapt to age‐related changes by redefining identity, independence, and participation. The findings further support inclusive conceptualisations of ageing well beyond a focus on impairment, function, and physical independence to encompass autonomy, purpose, and participation. These findings demonstrate that independence is not lost but redefined, so that independence is characterised by the capacity to participate in meaningful roles, rather than total self‐sufficiency. The study also highlights the importance of health literacy, system literacy and self‐advocacy as key determinants of ageing well, enabling people with SCI to navigate complex systems, overcome barriers and access supports to maintain participation and quality of life.

## 5 Conclusion

Ageing with SCI sustained before age 65 is shaped by three key determinants: funding; health and system literacy, including self‐advocacy; and meaningful participation. When disability funding meets basic needs, and individuals can navigate systems and advocate effectively, they are better able to focus on valued engagement in work, leisure, and social life. Barriers to ageing well include personal factors (physical changes, adjustment to functional changes, and adaptation to social change) and environmental factors (resource availability, access to skilled and knowledgeable health services, and societal attitudes towards disability and ageing). As the population ageing with SCI grows, it behoves us to support policy reform to ensure equitable resourcing, strengthen education for health professionals and people with lived experience, and promote meaningful participation to enable people to realise their potential to age well.

## Author Contributions

**Emma Tan:** conceptualization, data curation, writing – original draft, methodology, investigation, project administration, writing – review and editing; resources, validation. **Lynette Mackenzie:** conceptualization, investigation, methodology, validation, writing – review and editing; supervision, resources. **Jacqueline Wesson:** conceptualization, investigation, methodology, validation, writing – review and editing, supervision, resources. **Shane Clifton:** conceptualization, investigation, methodology, validation, writing – review and editing, supervision, resources.

## Ethics Statement

Ethics approval was provided by the University of Sydney Human Research Ethics Committee (2024/HE000461).

## Consent

Informed consent was obtained from all participants prior to participation in the study.

## Conflicts of Interest

The authors declare no conflicts of interest.

## Permission to Reproduce Material From Other Sources

No material from other sources has been reproduced in this study.

## Supporting information


Supporting File

## Data Availability

The qualitative data generated during this study are not publicly available due to the risk of participant identification, but may be available from the corresponding author on reasonable request, subject to ethics approval.
