# Supplementary material for: ‘From Superman to Barely Can:’ The Experience of Ageing for Australians With Spinal Cord Injury Sustained Below the Age of 65—A Qualitative Study
Source: Health Expect. 2026 Jul 28;29(4):e70787. doi: 10.1111/hex.70787 (PMC13411794; doi:10.1111/hex.70787)
Supplement: Supplementary file 1 — Supporting File [file HEX-29-e70787-s001.docx]

# Appendix 1: Consumer Advisory Panel Demographics

| Participants with SCI (n=8) |  |
| --- | --- |
| Age (years):   - Average - Standard deviation - Range | 65.5  9.4  53-81 |
| Gender:   - Female - Male | 3  5 |
| Level of Injury:   - Paraplegia - Tetraplegia | 4  4 |
| ASIA Score:   - A (Complete) - B (Incomplete) - D (Incomplete) - Unknown | 3  1  2  2 |
| Time Since Injury (years):   - Average - Standard deviation - Range | 23.2  16.5  5-49 |
| Location:   - Rural - Regional - Metropolitan | 1  2  5 |
| Carers (n=1)   - Female - Metropolitan | 1  1 |

# Appendix 2: Interview Schedule

Note: Interview questions are in italics. Themes to further prompt the interviewer are below each interview question in standard text.

**People with Lived Experience (Injury sustained < 65 years of age)**

Theme: Experience of ageing

1. *Please describe your experience of ageing or any changes you have experienced as you have got older?*

- Description of changes over time
- How changes have affected life roles

1. *How have your choices and agency over everyday roles and activities changed as you grow older?*

Theme: Support

1. *What support have you found useful to assist with the changes you have experienced?*

- Nature of support required and provided
- Experience of peer support

1. *Please tell me about any areas where you need more support*

- Additional support required and why

1. *Who do you see to help look after your health?*

- How health professionals are accessed
- What does healthcare look like for people with SCI
- Health professional knowledge of ageing with SCI

1. *You receive …… funding to assist with providing the supports you need for your SCI. What does this cover for you?*

- Adequacy and coverage of funding

Theme: Information Provision

1. *What kinds of things are there that you now know about ageing that you wish you knew earlier?*
2. *What sorts of information about ageing with SCI do you have? How did you find out this information?*

- Access to relevant information.

1. *What is the best way to communicate new information to people ageing with SCI?*

Theme: Perception of ageing

1. *People speak about their experience of discrimination from both a disability and age perspective. Is this something you have experienced? If so, could you please share some examples?*
2. *What are your biggest concerns about getting older? Why?*

**Carers / Support Workers:**

Theme: Experience of ageing

1. *What changes have you observed in the person that you care for as they get older?*

- Description of changes over time
- How changes have affected life roles

1. *How have their choices and agency over everyday roles and activities changed as they grow older?*

Theme: Carer experience

1. *What support do you think that they have they found useful to assist with the changes they have experienced?*

- Nature and amount of support required and provided

1. *Has caring for a person with SCI as they age changed the role of carer for you? How?*

- Level and nature of assistance provided and required over time
- How person is coping with caring role over time
- Support for carers

Theme: Support

1. *What are the areas where the person you care for needs more support? If so, what are these areas?*

- Additional support required and why

1. *Who does the person that you care for see to help them to look after their health?*

- How health professionals are accessed
- What does healthcare look like for people with SCI
- Health professional knowledge of ageing with SCI

1. *The person that you care for receives …… funding to assist with providing the supports they need for their SCI. What does this cover?*

- Adequacy and coverage of funding

Theme: Information Provision

1. *What kinds of things are there that you now know about ageing and SCI that you wish you knew earlier?*
2. *What sorts of information about ageing with SCI do you have? How did you find out this information?*

- Access to relevant information.

1. *What is the best way to communicate new information to carers so that you can better support people ageing with SCI?*

Theme: Perception of ageing

1. *People speak about their experience of discrimination from both a disability and age perspective. Has the person you care for been affected by discrimination? If so, could you please share some examples?*
2. *What are your biggest concerns for the person that you care for as they age? Why?*

# Appendix 3: Consolidated criteria for reporting qualitative studies (COREQ): 32-item checklist

Developed from:

Tong A, Sainsbury P, Craig J. Consolidated criteria for reporting qualitative research (COREQ): a 32-item checklist for interviews and focus groups. International Journal for Quality in Health Care. 2007. Volume 19, Number 6: pp. 349 – 357

| **Item No** | | **Guide Questions/Description** | **Reported on Page #** |  |
| --- | --- | --- | --- | --- |
| **Domain 1: Research team and reflexivity** | | | |  |
| **Personal Characteristics** | | | |  |
| 1. Interviewer/ facilitator | | Which author/s conducted the interview or focus group? | 7 |  |
| 2. Credentials | | What were the researcher’s credentials? E.g., PhD, MD | 4-5 |  |
| 3. Occupation | | What was their occupation at the time of the study? | 4-5 |  |
| 4. Gender | | Was the researcher male or female? | 4-5 |  |
| 5. Experience and training | | What experience or training did the researcher have? | 4-5 |  |
| **Relationship with participants** | | | |  |
| 6. Relationship established | | Was a relationship established prior to study commencement? | 6 |  |
| 7. Participant knowledge of the interviewer | | What did the participants know about the researcher? e.g. personal goals, reasons for doing the research? | 6 |  |
| 8. Interviewer characteristics | | What characteristics were reported about the interviewer/facilitator? e.g. Bias, assumptions, reasons and interests in the research topic | 4-5 |  |
| **Domain 2: study design** | | |  |  |
| **Theoretical framework** | | |  |  |
| 9.Methodological orientation and Theory | What methodological orientation was stated to underpin the study? e.g. grounded theory, discourse analysis, ethnography, phenomenology, content analysis | 7 |  |  |
| **Participant selection** | | |  |  |
| 10. Sampling | How were participants selected? e.g., purposive, convenience, consecutive, snowball | 7 |  |  |
| 11. Method of approach | How were participants approached? e.g., face-to-face, telephone, mail, email | 7 |  |  |
| 12. Sample size | How many participants were in the study? | 7 |  |  |
| 13. Non-participation Setting | How many people refused to participate or dropped out? Reasons? | 7 |  |  |
| 14. Setting of data collection | Where was the data collected? e.g., home, clinic, workplace | 7 |  |  |
| 15. Presence of nonparticipants | Was anyone else present besides the participants and researchers? | 7 |  |  |
| 16. Description of sample | What are the important characteristics of the sample? e.g. demographic data, date | 7-8 |  |  |
| **Data collection** | | |  | No |
| 17. Interview guide | Were questions, prompts, and guides provided by the authors? Was it pilot tested? | Appendix 2 |  |  |
| 18. Repeat interviews | Were repeat interviews carried out? If yes, how many? | 7 |  |  |
| 19. Audio/visual recording | Did the research use audio or visual recording to collect the data? | 7 |  |  |
| 20. Field notes | Were field notes made during and/or after the interview or focus group? | 7 |  |  |
| 21. Duration | What was the duration of the interviews or focus group? | 7 |  |  |
| 22. Data saturation | Was data saturation discussed? | 7 |  |  |
| 23. Transcripts returned | Were transcripts returned to participants for comment and/or correction? | 7 |  |  |
| **Domain 3: analysis and findings** | | |  |  |
| **Data analysis** | | |  |  |
| 24. Number of data coders | How many data coders coded the data? | 7 |  |  |
| 25. Description of the coding tree | Did the authors provide a description of the coding tree? | N/A |  |  |
| 26. Derivation of themes | Were themes identified in advance or derived from the data? | 7 |  |  |
| 27. Software | What software, if applicable, was used to manage the data? | 7 |  |  |
| 28. Participant checking | Did participants provide feedback on the findings? | 7 |  |  |
| **Reporting** | | |  |  |
| 29. Quotations presented | Were participant quotations presented to illustrate the themes/findings? Was each quotation identified? e.g., participant number | 7-24 |  |  |
| 30. Data and findings consistent | Was there consistency between the data presented and the findings? | 7-30 |  |  |
| 31. Clarity of major themes | Were major themes clearly presented in the findings? | 7-30 |  |  |
| 32. Clarity of minor themes | Is there a description of diverse cases or a discussion of minor themes? | 7-30 |  |  |

# Appendix 4: Participants’ recommendations for information provision

| **Provider** | **Method** | **Comments** |
| --- | --- | --- |
| Peer support |  |  |
| - Formal | SCI organisations / mentors | No formal peer support programs for ageing |
| - Informal | Family/friend networks |  |
|  | Others with lived experience |  |
|  | Social media |  |
|  | Websites |  |
|  |  |  |
| Service Providers |  |  |
| - Health professionals | Structured workshops and programs | Face-to-face, online, information sessions, written resources |
|  | During therapy sessions |  |
|  | Ad-hoc discussions |  |
|  | Information phone-line | One-on-one support when required |
| - SCI organisations | Structured workshops and programs | Face-to-face, online, information sessions, websites, written resources |
| - Equipment suppliers | Information and advice on assistive technology/pragmatic solutions | Face-to-face, online, information sessions, websites, written resources |
| Note: |  |  |
| - Information is received most effectively at the time that it is relevant for the participant and when they are ready to hear it | | |

# Appendix 5: Activities and participation reported by study participants

| **Participation** | **Examples** |
| --- | --- |
| *Employment* |  |
| - Paid | Own business |
|  | Working for a company non-government organisation |
|  | Teaching/lecturing |
| - Voluntary | Fundraising |
|  | Community organisations. eg. Scouts |
| - Informal | Caring for grandchildren |
| *Advocacy* | Peer support |
|  | Mentoring |
|  | Advocacy committees |
|  | Policy advisor roles |
|  | Research involvement |
| *Social engagement* |  |
| - Informal | Socialising with friends/family |
| - Formal | Clubs (Bridge, mahjong) |
|  | Social groups |
| *Leisure* |  |
| - Exercise | Kayaking |
|  | Cycling |
|  | Gym |
|  | Pickleball |
|  | Surfing |
|  | Swimming |
|  | Yoga |
| - Creative | Ceramics |
|  | Painting |
|  | Attending theatre/opera |
|  | Library |
|  | Building models |
|  | Photography |
| - Sedentary | Watching TV |
|  | Reading |
| - Other | Birdwatching |
|  | General outings |
|  | Looking after pets |
| *Education* |  |
| - Formal | Courses |
|  | Tertiary study |
| - Informal | Own research on various topics, including assistive equipment |
